# Nano-Level Damage Characterization of Graphene/Polymer Cohesive Interface under Tensile Separation

**DOI:** 10.3390/polym11091435

**Published:** 2019-09-02

**Authors:** S. S. R. Koloor, S. M. Rahimian-Koloor, A. Karimzadeh, M. Hamdi, Michal Petrů, M. N. Tamin

**Affiliations:** 1 School of Mechanical Engineering, Universiti Teknologi Malaysia, Johor Bahru 81310, Malaysia; 2 Institute for Nanomaterials, Advanced Technologies and Innovation, Technical University of Liberec, Studentska 2, 461 17 Liberec, Czech Republic; 3 Molecular Simulation Research Laboratory, Department of Chemistry, Iran University of Science and Technology, Tehran 16846, Iran; 4 Centre of Advanced Manufacturing and Material Processing, Department of Mechanical Engineering, Faculty of Engineering, University of Malaya, Kuala Lumpur 50603, Malaysia; 5 Chancellory Office, National University of Malaysia, Bangi 43600, Selangor, Malaysia

**Keywords:** adhesives, cohesive zone model, finite element method, graphene-polymer nanocomposite, graphene/polymer interface, molecular dynamics, regressive softening law

## Abstract

The mechanical behavior of graphene/polymer interfaces in the graphene-reinforced epoxy nanocomposite is one of the factors that dictates the deformation and damage response of the nanocomposites. In this study, hybrid molecular dynamic (MD) and finite element (FE) simulations of a graphene/polymer nanocomposite are developed to characterize the elastic-damage behavior of graphene/polymer interfaces under a tensile separation condition. The MD results show that the graphene/epoxy interface behaves in the form of elastic-softening exponential regressive law. The FE results verify the adequacy of the cohesive zone model in accurate prediction of the interface damage behavior. The graphene/epoxy cohesive interface is characterized by normal stiffness, tensile strength, and fracture energy of 5 × 10^−8^ (aPa·nm^−1^), 9.75 × 10^−10^ (nm), 2.1 × 10^−10^ (N·nm^−1^) respectively, that is followed by an exponential regressive law with the exponent, *α* = 7.74. It is shown that the commonly assumed bilinear softening law of the cohesive interface could lead up to 55% error in the predicted separation of the interface.

## 1. Introduction

The ever-increasing demands for the usage of graphene-reinforced polymer composites in advanced instruments such as gas sensors, electrodes for batteries, solar cells, etc., require in-depth mechanical characterization of these nanomaterials [1,2,3]. Graphene nanostructure is a two-dimensional sheet of carbon atoms in a honeycomb hexagonal arrangement, which have shown exceptional potential for enhancing the thermal, mechanical, and electrical properties of polymers [4,5,6]. The excellent properties of the graphene sheets have motivated researchers to further explore the behavior of this nanostructure and its composition with polymers, which require new development of experimental methods, analytical approach, and numerical investigation [2,7,8,9,10]. The composition of graphene and polymer creates a nanocomposite with excellent mechanical properties, which is derived not only from the properties of the graphene and the polymer, but also from the interaction of these two constituent materials [2,8,11,12]. The load transfer between the reinforcement and the matrix in a composite is strongly affected by the interface between the two phases [13,14,15]. Therefore, the interface properties have a significant influence on the global behavior of nanocomposite materials, and should be considered in the design and simulation of the nanocomposite structures [14,15,16,17].

While numerous investigations have been performed on the mechanical characterization of the graphene, polymers, and graphene-polymer nanocomposites [4,14,18,19,20,21,22], a limited study was reported on the behavior of the graphene/polymer interface [10,16,23]. The behavior of the graphene/polymer interface is traceable to the atomic interactions between the two materials, thus could be quantified through simulations at the atomic level [10]. Among the various atomistic approaches, molecular dynamics (MD) and molecular structural mechanics (MSM) have been used to simulate the physical and mechanical behaviors of graphene-polymer nanocomposites [16,21,24,25,26]. In addition, the atomistic methods have been combined with the continuum-level approaches through multiscale modelling to quantify the mechanical response of nanocomposite materials [8,27,28]. In the continuum-based approach, the mechanical behavior of graphene-polymer nanocomposite is calculated using the multiscale models with random distribution of graphene structures in the polymer matrix [14,22,28,29]. In these models, the graphene/polymer interface was simulated either as perfectly bonded [23], using a spring model that behaves according to Lennard–Jones (L-J) potential [28], with the effective interface layer model [22,30], using a linear spring model for imperfect interface [14], or with the cohesive imperfect zone model [29]. The embedded graphene in the graphene-polymer nanocomposite exhibits different mechanical properties compared to the isolated one because the atomic interactions cause the formation of an imperfect bond between the polymer and the graphene [8]. Different studies on graphene- and nanocarbon fiber-reinforced polymer matrix composites reported a significant variation in the mechanical properties of the materials due to the Van der Waals interactions of the nanostructure with the surrounding polymer atoms during the curing process [8,19,31]. Such interaction creates a distinct interface between the nanostructured graphene and polymer phases, as identified by an irregular distribution of the polymer with an asymmetric density around the nanostructured graphene.

The cohesive zone model (CZM) is often used to simulate the elastic-damage behavior of the interface in composites at different length scales [13,16,29,32,33,34]. In the cohesive zone modelling, the interface behaves with linear elastic response to damage initiation at the maximum stress level, followed by damage evolution as represented by various forms of the softening laws [33,35,36,37,38]. In MD simulation, the cohesive law defines the atomic interaction at the micro-/nano-interface. It has been employed in the simulation of the deformation and fracture of graphene grain boundary [34], perfectly flat graphene sheet interacting with polyethylene [16], and embedded-atom method potentially for interface behavior of planar, tilt nano-grain boundary interface [39]. In addition, the hybrid MD-finite element (FE) simulation has been used to simulate the atomistic mechanism of the grain-boundary debonding of solid materials using the cohesive law [40]. The research has demonstrated the flexibility of the CZM to capture the linear-nonlinear behavior of the interface in single- and multi-phase materials, and at different length scales.

The objective of this study is to establish an accurate methodology for predicting the properties and mechanical response of the graphene/polymer interface in graphene-reinforced epoxy nanocomposites employing both the MD and FE simulation procedures. In the modeling process, the formation of chemical bonding at the graphene/epoxy interface during the curing of the nanocomposite is simulated using the MD approach. An in-house code is developed to simulate the polymer cross-linking process during the curing of the nanocomposite. Consequently, the characteristic of the graphene/epoxy cohesive interface under the tensile separation loading is established. The resulting properties of this nanoscale cohesive interface are then prescribed for the cohesive elements of the FE simulation approach. This enables the accurate FE prediction of the properties and the mechanics of deformation of the graphene-reinforced epoxy nanocomposites in the multiscale simulation.

## 2. Computational Methods

### 2.1. Molecular Dynamics Simulation

The elastic and damage behaviors of the graphene/polymer interface in the nanocomposite were determined through the MD simulation of the nanocomposite. For this purpose, a representative volume element (RVE) of the graphene-polymer nanocomposite with the specifications provided in Table 1, was created for modelling the curing process of the polymer matrix in the presence of the graphene. The simulation of the polymer curing process and the tensile loading of the nanocomposite using the MD modelling are described in the following subsections.

#### 2.1.1. Cross-Linking of the Polymer during the Curing Process

**Figure 1:**
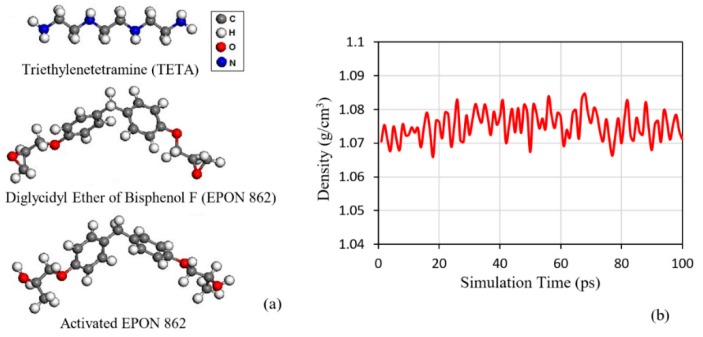
(**a**) Molecular structure of the polymer matrix used in the molecular dynamics (MD) simulation, (**b**) variation of RVE density against time after the initial isothermal-isobaric ensemble (NPT) equilibration.

The initial molecular model of the polymer was created in PACKMOL software. The model was then submitted to the large scale atomic/molecular massively parallel simulator (LAMMPS) software to model the curing process of the polymer [41,42]. The polymer matrix consists of Diglycidyl Ether of Bisphenol F (EPON 862) and Triethylenetetramine (TETA) as the resin and hardener respectively, with a stoichiometric ratio of 3:1. In an epoxy reaction with the hardener group, the lone pair of nitrogen atoms is attached to the open epoxy rings. However, such an in-situ reaction could not be modelled in the classical MD simulation based on Newton’s laws, which requires the chemical bonds and the subsequent atomic structures to be defined prior to the simulation and remain unchanged until the end of the process. Therefore, an algorithm was developed to simulate the reaction between hardener and EPON through the cross-linking process. In this algorithm, the epoxy rings of the EPON molecule were opened to form activated EPON 862, to simplify this reaction before packing, as illustrated in Figure 1a. Then, the EPON and TETA molecules with optimized geometries were loaded with the ratio of 390:130 inside the simulation box. This procedure was done at the density of 0.8 g/cm^3^ in order to restrict the formation of unreal configuration and huge forces which occur due to the ring spearing and atoms overlapping [43,44]. The density of the molecular system was increased to the real density through the initial equilibration level by the isothermal-isobaric ensemble (NPT) [23,45,46]. Figure 1b shows the variation of the RVE density with respect to the simulation time after the initial equilibration. The curing process was performed subsequently on this equilibrated molecular system.

In the modelling of the nanocomposites RVE, the graphene sheet was inserted along the x-y plane such that its center-of-mass is placed on the center of the simulation box, while the polymer molecules were positioned around the graphene sheet. Subsequently, the curing process was applied through the static cross-linking procedure [43,47,48]. This was executed through a written FORTRAN programming code, and compiled in the Linux platform. The code consists of the following steps:

1. It was assumed that the primary and secondary amines have the same reactivity with the reaction cutoff distance of 5 Ǻ.
2. The system was checked for the bond formation at every 50 ps after each equilibration.
3. The constants in the equation of the newly-formed bonds were decreased in the first equilibration level and progressively elevated to the real values [47].
4. The outputs were examined to update the topology parameters according to the new bonds.
5. An annealing process was implemented to release the residual stresses. In this process, the temperature was increased to 450 K and then cooled to 300 K gradually over 2 × 106 time-steps.
6. Steps 2 through 5 were iterated to obtain about 80% cross-linking.
7. Finally, the last NPT equilibration in an interval time of 3 ns was applied to the RVE.

The MD simulation procedure of the curing process is verified by comparing the predicted elastic properties of the epoxy polymer with those of measured values. In this process, an RVE of the epoxy polymer is created and cured to establish the intrinsic properties. The cured epoxy model is then loaded in tension to establish the force-displacement response in the elastic range, thus the elastic properties of the polymer could be determined. The details of the modelling steps are discussed elsewhere [31]. The close comparison of the predicted and measured properties [49,50], as listed in Table 2, serves to validate the MD modelling of the curing process.

#### 2.1.2. Tensile Separation Process

The MD simulation of the graphene-polymer nanocomposite under tensile loading was performed using LAMMPS software [42] with a pair cut-off distance of 12 Ǻ and a time step size of 1 fs. The graphene was modelled based on the Adaptive Intermolecular Reactive Empirical Bond Order (AIREBO) Potential of Stuart as [51]:

(1) $$V=\frac{1}{2}\sum_{i}\sum_{i\neq j}\left[V_{ij}^{REBO}+V_{ij}^{LJ}+\sum_{k\neq i,j}\sum_{l\neq i,j,k}V_{kijl}^{TORSION}\right]$$

where, $V_{ij}^{REBO}$ is the Reactive Empirical Bond Order (REBO) potential by Brenner, $V_{ij}^{LJ}$ defines longer-ranged interactions (2 < r < cutoff) by an equation similar to standard L-J potential, and $V_{kijl}^{TORSION}$ describes different explicit 4-body dihedral angles [52].

The polymer matrix and the Van der Waals interactions between the graphene and polymer atoms were simulated based on the ab initio polymer consistent force field (PCFF) [23,53,54]. The PCFF force field is able to accurately represent the interactions between the sp^2^ carbon atoms of graphene and all of the polymer atoms in the condensed graphene-polymer nanocomposites [23,54]. In the PCFF force field, the non-bonded Van der Waals interaction between the same type atoms (denoted by *ii*) is described using the L-J potential as:

(2) $$V=\varepsilon \;\left[2{\left(\frac{\sigma }{r}\right)}^{9}-3{\left(\frac{\sigma }{r}\right)}^{6}\right]\;\;r<r_{c}$$

where, V is the interaction energy, σ represents the distance at which the interatomic interactions are zero, ε is the depth of the potential well, and r and $r_{c}$ are inter atomic and cutoff distances, respectively. In computational chemistry, the interaction energy between two dissimilar non-bonded atoms is obtained using the mixing rule [55]. Therefore, the values of ε and σ for two atoms of different types of *i* and *j* were calculated based on the sixth-power mixing rule, as:

(3) εij=2(εiεj)(σi3σj3)σi6+σj6 σij=((σi6+σj6)2)16

**Figure 2:**
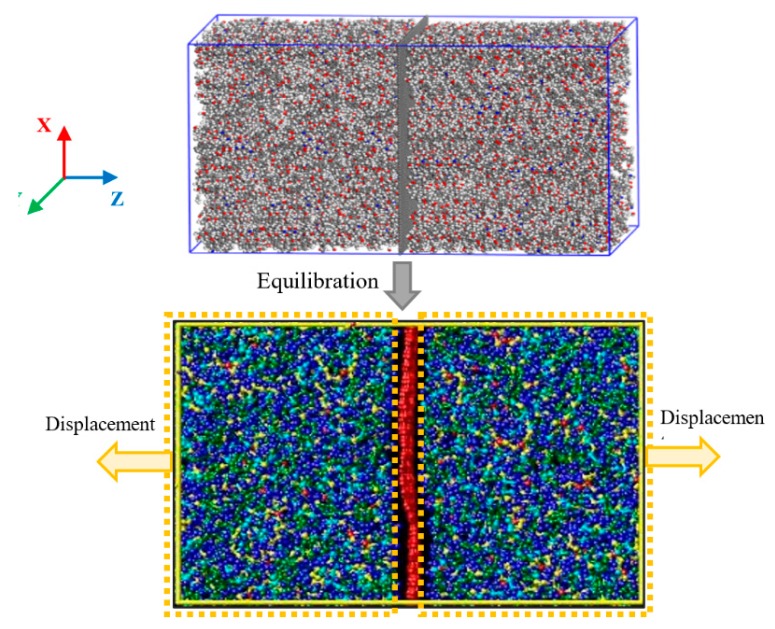
The simulation box containing long embedded graphene with the applied displacement of the nanocomposite.

The RVE was modelled as a long structure of embedded graphene in the epoxy matrix, with a specified periodic boundary condition [31], as shown in Figure 2. In this model, the temperature was equilibrated at 300 K and the pressure was adjusted to one atmosphere in all directions, using the Nose–Hoover style of non-Hamiltonian equations of motion. Consequently, the pressure was maintained uniform and equal in the x- and y-directions (graphene in-plane directions), and independently in the z-direction. The MD simulation of the nanocomposite RVE is used to establish the evolution of stress with the tensile deformation of the graphene/polymer interface. The polymer atoms of the model, as shown in Figure 2, are displaced along the z-direction, such that the distance between any two layers of the atom in the x-y-plane remains fixed. The atoms in a plane are free to move in the x-y-plane. Under this periodic boundary condition, the RVE box dimension is displaced in the Z-direction with a rate of 10^−7^ ns^−1^, the other faces were maintained under one atmospheric pressure [56,57].

Following each loading step, the stress components, *τ_pq_*, were calculated using Virial expression as [58,59]:

(4) $$\tau_{pq}=-\;\frac{1}{\upsilon }\left[\left(\overset{N}{\underset{i=1}{\sum }}m_{i}\left(\nu_{ip}\;\nu_{iq}\right)\right)+\;\left(\underset{i<j}{\sum }r_{ijp}f_{ijq}\right)\right]$$

where, *m* and ν are the mass and velocity of atom *i* respectively, υ is the total volume of the RVE, and f denotes the force between atoms with *i* and *j* indices. The vector *τ_pq_* represents the stress components *τ_xx_*, *τ_yy_*, *τ_zz_*, *τ_xy_*, *τ_xz_*, and *τ_yz_*.

### 2.2. Continuum Mechanics Simulation

The MD model of the graphene-polymer nanocomposite, as shown in Figure 2, is employed in the continuum mechanics analysis through the FE simulation. The graphene/polymer interface was modelled with the cohesive behavior using the cohesive element.

#### 2.2.1. Cohesive Zone Model

**Figure 3:**
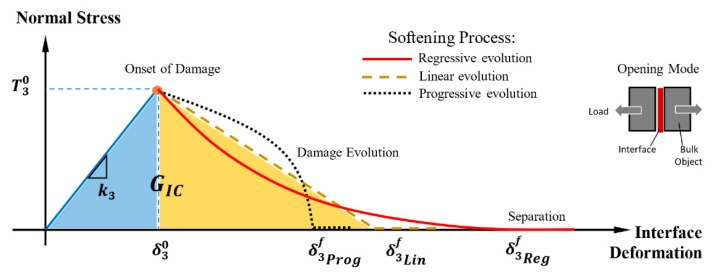
The cohesive softening law to describe interface behavior in the tensile separation mode.

The mechanical behavior of the interface between two continuum bodies can be simulated using the CZM with a bilinear traction-separation law for single- (normal or shear) and mixed-mode loading conditions [33,35]. Under the tensile loading, the elastic-softening behavior of the interface is illustrated in Figure 3. The interface material point behaves linearly until damage initiation occurs at the maximum traction. This is followed by the softening process until the tensile separation of the material point. The softening process could be described by a gradual decreasing stress with the applied displacement through a linear, regressive, or progressive curve, as illustrated in Figure 3.

The initial linear elastic behavior of the interface is described by:

(5) $$\left\{\begin{array}{c} t_{3}\\ t_{1}\\ t_{2} \end{array}\right\}=\;\left[\begin{array}{ccc} k_{3} & 0 & 0\\ 0 & k_{1} & 0\\ 0 & 0 & k_{2} \end{array}\right]\;\left\{\begin{array}{c} \delta_{3}\\ \delta_{1}\\ \delta_{2} \end{array}\right\}$$

where, $t_{i}$ and *δ_i_* are the traction and separation at an interface point, and indices 3, 1, and 2 refer to mode I (normal) load, mode II, and mode III (shear) loadings, respectively. The parameter *k_i_* represents the cohesive stiffness of the interface in the respective load direction. In this study, the pure tensile loading mode was considered, thus the traction-displacement relation could be simplified to $t_{3}=k_{3}\delta_{3}$.

#### 2.2.2. Damage Initiation and Propagation Criterion

A quadratic stress-based criterion was used to indicate the damage initiation in the mixed mode condition as:

(6) $$\sqrt{{\left(\frac{t_{3}}{T_{3}^{0}}\right)}^{2}+{\left(\frac{t_{2}}{T_{1}^{o}}\right)}^{2}+{\left(\frac{t_{1}}{T_{2}^{o}}\right)}^{2}}=d$$

where, $T_{i}^{0}$ represents the cohesive strength, and  d is the damage initiation variable. The variable *d* varies from “zero” for the pristine interface to “one” indicating the onset of interface of damage. In the pure tensile mode, Equation (6) was simplified to ${\left(\frac{\left\langle t_{3}\right\rangle }{T_{3}^{0}}\right)}^{2}=d$, The total energy dissipated during the elastic-softening deformation of the interface, as represented by the area under the traction-displacement curve, represents the critical strain energy release rate, ($G_{\mathrm{IC}}$, Figure 3). This is also representing the fracture energy of the interface which is fixed for any softening law being used for the analysis. The strain energy release rate, *G_i_*, in each loading mode could be calculated by the following equation:

(7) $$G_{i}=\int_{0}^{\delta_{i}{}_{m}^{f}}t_{i}d\delta_{i}\;\;\;\;\;\;\;\;\;i=1,\;2,\;3$$

where, $\delta_{i}{}_{m}^{f}$ is the relative displacement at failure. This displacement is a dependent parameter to the type of interface softening process, as shown in Figure 3. The graphene-polymer interface behavior in this study was computed using the regressive softening law in an exponential decay form. The damage propagation variable, $d_{p}{}_{i}$ was defined as [60]:

(8) $$d_{p}{}_{i}=1-\left(\frac{\delta_{i}^{0}}{\delta_{i}}\right)\left(1-\frac{1-e^{\left(-\alpha \left(\frac{\delta_{i}-\delta_{i}^{0}}{\delta_{i}^{f}-\delta_{i}^{0}}\right)\right)}}{1-e^{\left(-\alpha \right)}}\right)\;i=1,2,\;3$$

where, *α* is a non-dimensional interface parameter that determined by the rate of damage evolution. This parameter was calculated from the exponential decay curve of the interface softening behavior through the MD simulation of the graphene-polymer interface.

#### 2.2.3. Finite Element Simulation

**Figure 4:**
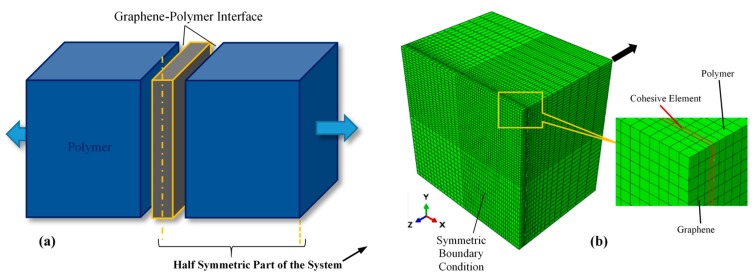
(**a**) Schematic view of the graphene-polymer system, and (**b**) finite element (FE) model of the half symmetric part of the nanocomposite system.

The geometrical model of the RVE consisting of a graphene sheet bonded by two blocks of epoxy polymer is shown in Figure 4a. The geometrical properties of the nanocomposite model are as provided in Table 1. The interface thickness was determined from the MD simulation of the curing process (see Section 3.1). Due to the symmetry of the RVE model, only one-half of the model with one-half thickness of the graphene layer was represented in the FE simulation. The graphene layer and the polymer block were discretized using continuum elements (8-node, linear, reduced integration elements (C3D8R)). A layer of 8-node cohesive elements (COH3D8) were employed to represent the graphene/epoxy interface, as illustrated in Figure 4b. A mesh convergence study was performed to ensure that the FE-calculated variables are independent of the size of the elements. This resulted in small-size elements in the region next to the interface and gradually larger elements away from the interface. In addition, the FE model was partitioned into different volumes, each discretized with a slightly different mesh density to avoid the effects of symmetrical mesh distribution on the computed results [61].

The hyper-elastic behavior of the epoxy and the properties of the graphene used in the FE simulation were reported elsewhere [8]. The properties of the CZM were obtained through the MD simulation (see Section 3.2), performed prior to the FE analysis. A symmetric boundary condition was assigned to the outer surface of the graphene later, as illustrated in Figure 4b. A reference point was identified at the centroid of the polymer block to “tie” all the elements of the polymer. A global displacement in the z-direction is prescribed for the reference point so as to reproduce identical loading conditions that were used in the MD simulation. The traction-displacement curve of the graphene/epoxy interface could then be extracted.

## 3. Results and Discussion

The computational results of the graphene-polymer nanocomposite model, performed in MD and FE simulation environments, are presented and discussed in the following subsections. The mechanics of deformation of the graphene/polymer interface under the tensile loading is described. The validation aspect of the FE simulation approach in capturing the physics of the interface failure process is deliberated.

### 3.1. Thickness of the Graphene and Graphene/Polymer Interface

**Figure 5:**
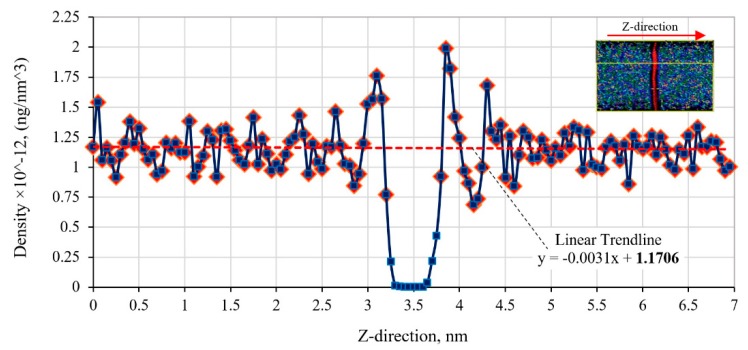
Variation of the epoxy polymer density along the RVE length of the graphene-polymer nanocomposite.

The density profile of the epoxy polymer across the cured polymer/graphene/polymer phases of the RVE model is shown in Figure 5. The average density of the epoxy is estimated at 1.17 × 10^12^ ng/nm^3^, as illustrated by the dashed line and without considering the density readings below 0.65 × 10^12^ ng/nm^3^. It is noted that a 0.3% variation of the average density of the epoxy is calculated over the sampling distance of 7 nm, particularly contributed by the higher density gradient next to the graphene/polymer interface. The polymer density diminishes to zero across a small gap to the location of the graphene. This density gradient indicates the non-uniform distribution of the polymer atoms in the nearby graphene/polymer interface.

The profile was used to establish the boundary of the graphene and polymer constituents. The RVE was segmented into equal spacing of 0.05 nm along the z-direction (across the graphene/polymer interface). A zero-density gap with a thickness of 0.40 nm was determined at the central location of the nanocomposite RVE. The graphene thickness was assumed to be similar to the distance between the graphene sheets in the graphite structure (0.34 nm) and located in the middle of the nanocomposite RVE. Thus, the graphene/epoxy interface thickness on each side of the graphene is determined as having the thickness of 0.03 nm. The epoxy matrix on each side of the graphene was considered as 3.3 nm-thick with the cross-sectional area of 4.52 × 4.54 nm^2^ (see Table 1) for the RVE. The calculated thickness of the graphene/epoxy interface will be used in the FE model of the RVE with finite-thickness cohesive elements.

### 3.2. Nanomechanical Behaviors of Graphene/Polymer Interface

**Figure 6:**
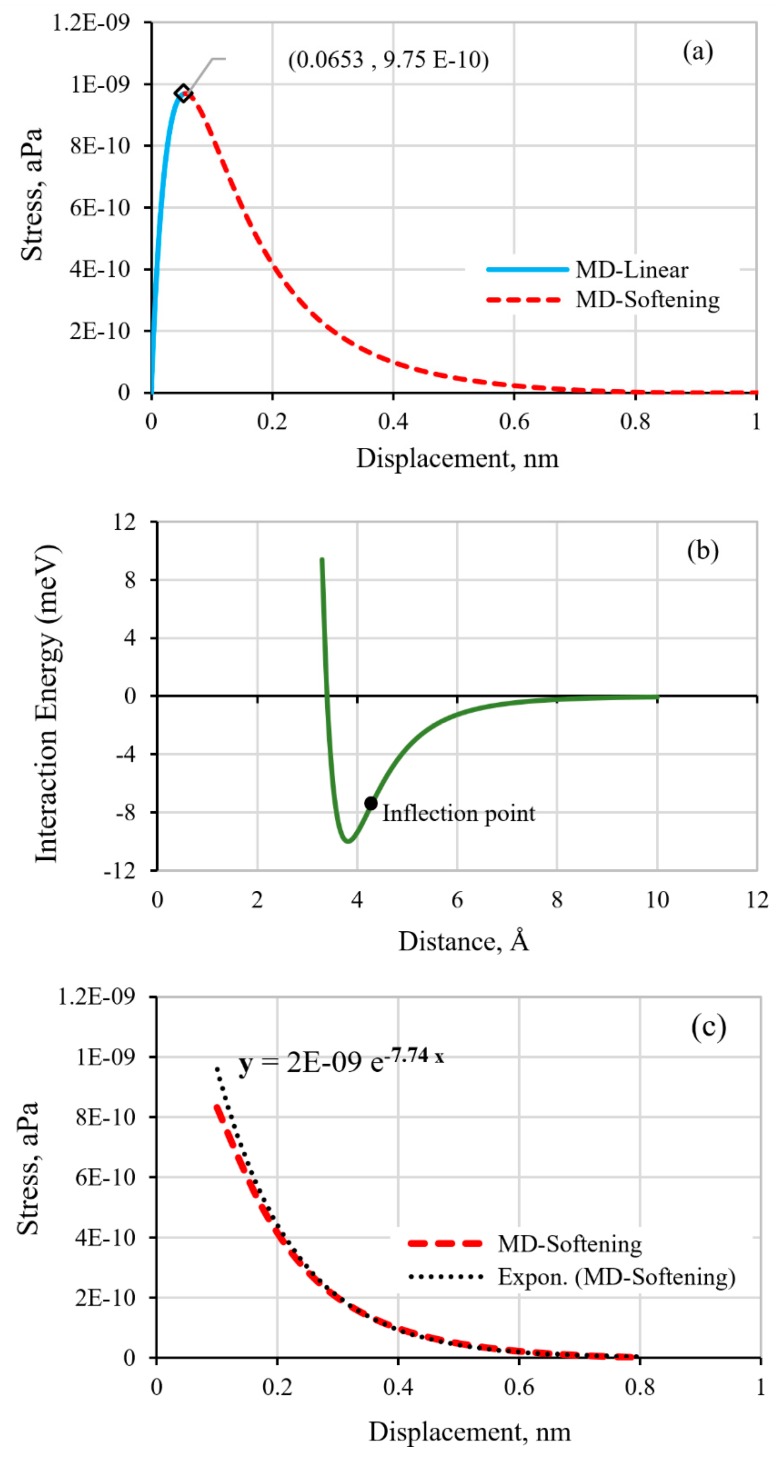
(**a**) The stress-displacement response, as calculated from the MD simulation, (**b**) interatomic energy-distance curve, and (**c**) the characteristic exponential decay softening law of the CZM.

The nanomechanical behavior of the graphene/epoxy interface, predicted using the MD simulation, is shown by the stress-displacement curve in Figure 6a. The results showed that, throughout the applied tensile loading (displacement-controlled), a non-linear increase in the interface stress to the peak level of 9.75 × 10^−10^ aPa is achieved. The stress decreases exponentially with the continuous displacement. A similar cohesive stress-displacement response with a nonlinear spring model was predicted for the interface of a pristine graphene/one-layer polymer using the MD approach [10,16]. At the atomic level, the interaction between the neighboring atoms could be described in terms of the interaction energy, as shown in Figure 6b, for two atoms with ε=3.4 Å and σ=10 meV. The interaction energy level is at a minimum (also known as the potential well) when the atoms are at their equilibrium distance apart, such that the cohesive (attractive) and repulsive forces between the atoms are balanced. Under the applied separation forces, the interaction energy decreases in an exponential manner, but the energy is restored upon unloading. If the loading continued, the separation energy would diminish to zero, denoting the atomic separation. The inflection point denotes the minimum interaction force as represented by the slope of the energy-distance curve (Figure 6b). Beyond this distance, the required interaction force of atomic separation that diminish exponentially could be interpreted as representing the degradation of the bond strength.

Analogously, while the initial deformation of the graphene/epoxy interface is elastic, beyond the displacement corresponding to the peak stress the decreasing stress-displacement curve also exhibits an exponential decay form, and constitutes of both recoverable and irrecoverable components (Figure 6a). Only when the tensile separation is large enough, the stress completely diminishes to cause the separation of the interface material point. The exponential decrease portion of the stress-displacement curve, as predicted by the MD simulation, is postulated to adequately represent the softening behavior of the cohesive graphene/epoxy interface at the continuum scale. Consequently, the softening characteristic of the CZM (see Figure 3) for the interface could be established based on the exponential decay curve, as illustrated in Figure 6c. The corresponding rate of the decay, as described in Equation (8), is characterized by the exponent, *α* = 7.74.

**Graphene/epoxy interface properties:** The elastic properties of the interface were also determined from the MD-calculated stress-displacement curve of Figure 6a. The interface tensile strength, $T_{3}^{o}$, is defined by the peak stress level while the corresponding displacement represents the value at the onset of interface damage, $\delta_{33}^{o}$. The initial tangential slope of the stress-displacement curve defines the penalty stiffness, *k*_3_, of the interface. The area bounded by the curve represents the critical strain energy release rate, *G_IC_*, for the tensile loading mode, which could be calculated by Equation (7). These properties along with the parameters of the exponential softening law are used to define the graphene/epoxy cohesive interface behavior for the FE simulation. The property values are listed in Table 3.

### 3.3. Response of the Graphene/Epoxy Interface Model

**Figure 7:**
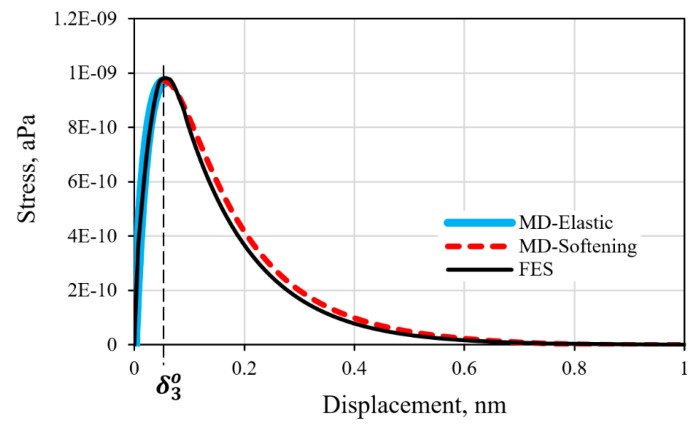
Comparison of the FE and MD stress-displacement response of the graphene/polymer interface.

The FE-calculated stress-displacement response of the graphene/epoxy cohesive interface using the properties listed in Table 3 is shown in Figure 7. The close correlation of the CZM response with the input response from the MD simulation, with less than 3% difference, served to verify that appropriate procedures were employed in developing the FE model. The deviation is likely due to the different types of boundary condition specified for the MD and FE model resulting in slightly different stress levels for the onset of damage in each case. The intrinsic exponential-decay cohesive response of the graphene/epoxy interface, as established by the MD simulation, is faithfully reproduced by the FE model.

**Figure 8:**
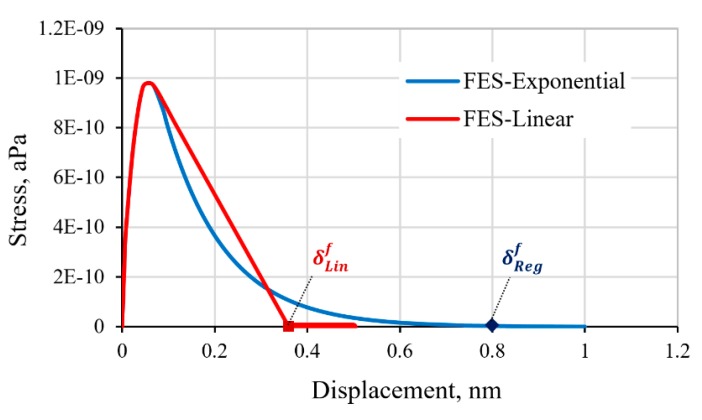
Comparison of the FE-predicted response of the cohesive interface with different softening laws.

In the absence of the MD-predicted exponential regressive behavior, a bilinear softening law is often employed as an estimate of the cohesive interface behavior. The response of the FE model with a bilinear softening law specified for the cohesive interface was examined. The CZM properties of tensile strength, penalty stiffness, and the critical Mode I strain energy release rate is maintained in the simulation to acknowledge the same physical interface. The resulting stress-displacement curves are compared in Figure 8. Results show that while insignificant differences of the elastic load-displacement response were displayed, the stress evolution during the damage process significantly differs. The bilinear softening law predicted a higher interface stress than the exponential regressive law by 25%, corresponding to the tensile displacement of 0.2 nm. In addition, the bilinear softening law would also predict an earlier separation of the cohesive interface. The interface separation is calculated at the displacement of 0.36 nm using the linear softening law, which corresponds to a 55% difference when compared to the prediction by the regressive softening rule of the interface at the displacement of 0.80 nm. Thus, the use of an inaccurate softening law and properties of the cohesive interface could lead to erroneous predicted properties, and the corresponding deformation mechanics and the failure process of the graphene-epoxy polymer nanocomposite.

**Figure 9:**
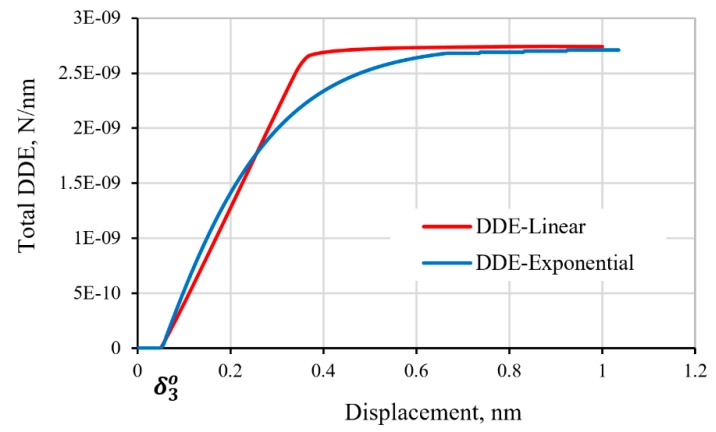
Evolution of the total damage dissipation energy (DDE) of the graphene/polymer interface with increasing tensile displacement.

The energy released by the graphene/epoxy interface during the interface failure process under the applied tensile loading is termed the damage dissipation energy (DDE). The evolution of the total DDE, for all the cohesive interface elements of the model with the two different softening laws of the cohesive interface, is compared in Figure 9. The DDE started to accumulate when the damage initiated at the attainment of the maximum load, corresponding to the damage initiation displacement, *δ_o_*. The calculated evolution of the DDE follows the prescribed form of the regressive softening process, as illustrated in the figure. The initial rate of the energy dissipation is faster for the cohesive interface with exponential regressive than that of the linear softening law, as reflected by the initial slope of the curve. The evolving DDE saturates to a constant level when reaching the separation of the graphene/epoxy cohesive interface.

## 4. Conclusions

The deformation and failure process of the graphene-polymer nanocomposites could be quantified through the FE simulation with the cohesive interface elements. The missing intrinsic properties and behavior of the cohesive interface could be determined from the MD simulation of the curing and subsequent loading of the nanocomposite RVE. This paper has demonstrated the procedures in establishing the intrinsic regressive softening law for the cohesive interface of the graphene/epoxy nanocomposites. Results show that the graphene/epoxy interface has a thickness of 0.03 nm, based on the density variation of the polymer across the graphene/epoxy interphase. Upon tensile loading of the RVE, an exponential-decay form of the cohesive interatomic forces was established. Analogously, the cohesive interface was described by the exponential regression law with the exponent, *α =* 7.74. The assumed bilinear softening law of the cohesive interface, commonly employed in the microscale FE simulation of the nanocomposite behavior, could lead to erroneous results when compared to the MD-calculated exponential regression law. This was demonstrated by the different traction-displacement curves and the evolution of DDE to tensile separation of the graphene/epoxy interface of the nanocomposite RVE. A 55% difference in the predicted displacement at the separation of the interface was calculated when the bilinear softening law was assumed, compared to that using the exponential regressive damage law of the cohesive interface.

## Figures and Tables

**Table 1:** The equilibrated representative volume element (RVE) specifications of the graphene-epoxy nanocomposite.

Configuration of RVE	Graphene Sheet Length (nm)	Box Volume (nm^3^)	Number of Epoxy Molecules	Number of Hardener Molecules	Density after Curing Process and Final NPT Equilibration (g/cm^3^)
Long	4.540 × 4.520	143.653	258	86	1.1865

**Table 2:** Properties of the epoxy polymer obtained from the MD simulation and experiments.

Parameter	MD Simulation	Experiments
**Density, g/cm^3^**	1.14	1.16
**Poisson’s ratio**	0.39	0.3–0.4
**Young’s modulus, GPa**	2.77	2.4–3.4
**Shear modulus, GPa**	1.03	1.0–1.5

**Table 3:** The elastic and damage properties of graphene/polymer cohesive interface.

Parameter	Symbol (Unit)	Value
**Tensile stiffness**	*k*_3_, (aPa·nm^−1^)	5 × 10^−8^
**Tensile strength**	$T_{3}^{0}$, (aPa)	9.75 × 10^−10^
**Displacement at damage initiation**	$\delta_{33}^{o}$, (nm)	0.0653
**Displacement at separation**	$\delta_{33}^{f}$, (nm)	0.8
**Exponent for the regressive softening law,** (Equation (8))	*α*	7.74
**Critical Mode I strain energy release rate**	*G_IC_*, (N·nm^−1^)	2.1 × 10^−10^
